# Importance of Considering Temporal Variations in Pulse Wave Velocity for Accurate Blood Pressure Prediction

**DOI:** 10.1007/s10439-025-03681-7

**Published:** 2025-02-06

**Authors:** Aditya Satishkumar Bantwal, Amit Kumar Bhayadia, Hui Meng

**Affiliations:** Department of Mechanical and Aerospace Engineering, University at Buffalo, Buffalo, NY 14260 USA

**Keywords:** Cuffless blood pressure, Pulse wave velocity, Pulse transit time, Fluid-structure interaction, Radial artery, Systolic

## Abstract

**Purpose:**

Continuous, cuffless blood pressure (BP) monitoring devices based on measuring pulse wave velocity (PWV) or pulse transit time (PTT) are emerging but are often plagued by large prediction errors. A key issue is that these techniques typically rely on a single PWV value, assuming a linear response and small arterial wall deformations. However, arterial response to BP is inherently nonlinear, with PWV varying over time [PWV(t)] by up to 50% during a cardiac cycle. This study evaluates the impact of assuming a single PWV on BP prediction accuracy.

**Method:**

Using a Fluid-structure Interaction (FSI) testbed, we simulate the radial and common carotid arteries with the Holzapfel–Gasser–Ogden (HGO) constitutive model to capture nonlinear arterial behavior under a pulsatile physiological blood flow. Pressure data from FSI simulation are used as the ground truth, while inner area A(t) and two PWV values, at diastole and systole, serve as inputs to BP prediction models. Two models are tested: one using a single PWV value, emulating existing PWV-based BP prediction methods; another using the two PWV values to account for PWV(t).

**Results:**

The single-PWV BP model produced prediction errors of 17.44 mmHg and 6.57 mmHg for the radial and carotid arteries, respectively. The model incorporating two PWV values reduced these errors by 90.6% and 96.8%, respectively.

**Conclusion:**

Relying on a single PWV in BP prediction models can lead to significant errors. To improve BP accuracy, future efforts should focus on incorporating PWV(t), or at least both diastolic and systolic PWV values, into these models.

## Introduction

Hypertension is called the “silent killer”. Showing almost no symptoms at an early stage, hypertension is often associated with the onset and development of major cardiovascular diseases such as heart disease and stroke [1, 2]. For preventing the onset of major cardiovascular events, it is crucial to continuously monitor BP. However, current BP measurement methods are not suitable for continuous monitoring of BP. The gold standard of BP measurement uses an endovascular arterial line. It is invasive and usually only applied in serious medical conditions [3]. In clinics and home environments, BP is measured using a sphygmomanometer or oscillometer [4]. These cuff-based devices require inflating the arm, which is cumbersome and uncomfortable. Furthermore, they cannot continuously provide immediate indications of sudden, rapid changes in BP. These issues of arterial line and cuff-based devices call for cuffless devices that are noninvasive, comfortably wearable for continual operation, and capable of continuously transmitting BP data to healthcare providers.

A commonly pursued approach to cuffless and continuous BP monitoring devices involves BP estimation using Pulse Wave Velocity (PWV) [3, 5–8], *i.e.*, the speed of blood pressure waves propagating in the artery, or equivalently, the time delay of a pulse wave over a known distance along the artery known as pulse transit time (PTT). This is measured by optical [9–13], ultrasonic [14–19], or piezoelectric sensors [20]. The underlying relationship between PWV and BP hinges on arterial compliance, which is influenced by BP. As blood pressure rises, arteries stiffen due to increased engagement of collagen fibers to support the higher load, thereby affecting PWV. Such approaches predict BP from PWV based on a mathematical model obtained either analytically with unknown coefficients being determined from subject-specific calibration [3, 21], or entirely from machine learning and regression [13, 22]. Since PWV is a noninvasively measurable quantity that does not require a cuff [20], the PWV-based BP prediction methods are promising for implementation as continuously wearable devices. However, despite plentiful investment into these technologies, the performance of current PWV-based BP efforts is not universally accepted [5, 13, 22]. They have shown large errors [3, 21, 22], ranging from 20 to 90% [3]. The cause for such universally large errors is not entirely clear, thus severely hindering further development of continuous BP monitoring technologies from PWV.

An often overlooked key fact in essentially all current PWV-based BP measurement approaches is that they rely on a single PWV value (*e.g*., [3, 6, 14, 15, 18–21, 23–27]). This implies that PWV is a constant within a cardiac cycle despite BP changing with time [28]. In a recent biomechanics study based on Fluid-Structure-Interaction (FSI) [28], we have shown that assuming a constant PWV is equivalent to assuming that the arterial wall behaves like a linear elastic material [28]. Such a linear elastic assumption is only appropriate for thin-walled arteries (thickness $\le$ 5–10% of inner radius [29]) undergoing small deformations (theoretically infinitesimal; practically $\le$ 5–10% of radius [29]). However, the arterial wall in vivo can undergo large, nonlinear deformations up to 30% of its radius [8, 29–33]. As a consequence, we showed that PWV can vary up to 50% within a cardiac cycle [28]. This finding is supported by in vivo studies in human subjects. For example, one study performed BP measurement in the radial artery using an invasive arterial line [11], showing that PWV increased with BP and displayed up to 50% variations within a pressure range of $\approx$ 40 mmHg. Another study focused on the carotid artery [33] and obtained PWV values from an empirical relation between BP and area, showing that PWV varied by 35% in a cardiac cycle.

PWV variation with time has been occasionally acknowledged in the literature [11, 16, 34, 35], but it has been mostly ignored when it comes to developing BP prediction methods using PWV. The assumption of a constant PWV in the existing BP prediction models may not have been intentional, given that current devices typically only offer a single time delay of a pulse waveform. Furthermore, many of the existing BP-PWV models [3, 6], are derived from the Moens–Korteweg–Hughes equation, which only requires a single PWV value, and this equation implicitly assumes linear arterial response [28].

Our objective is to assess the impact of using a single PWV on the BP prediction accuracy, compared against considering a time-varying $PWV(t)$. To that end, we use a well-controlled numerical testbed based on FSI simulations of an artery subjected to a physiological pulsatile flow. FSI is the most appropriate computational tool for investigating the physics governing the relationship between BP and PWV, since it captures the dynamic interactions between blood flow and the arterial wall during pulse wave propagation. The arterial blood flow dynamics are simulated under physiological flow rate and pressure boundary conditions, along with a nonlinear material model allowing the arterial wall to undergo large, nonlinear deformations [36]. Simulating the arterial FSI under these realistic conditions creates controlled scenarios, providing a unique opportunity to not only understand the physics underlying BP and PWV, but also test different BP prediction models systematically. Such levels of flexibility are lacking during in vitro experiments for testing PWV-based BP prediction that typically employ an arterial tube phantom that behaves as a linear elastic material [26], and during in vivo tests, whose accuracy can be questioned when the reference BP is acquired at a different arterial location than the artery where BP is predicted [26, 33]. Furthermore, such investigation is not feasible in population-based studies, where it would be challenging to control and understand all the variables that affect the relationship between BP and PWV.

In this study, our primary artery of interest is the radial artery. Even though there have not been many biomechanical or clinical studies of the radial artery, we choose to investigate BP prediction in this artery as it is a common site for wearable cuff-less sensors with the potential of implementing BP monitoring on a smartwatch. An additional artery we consider in this study is the common carotid artery (CCA). It is widely recognized that carotid artery BP is more strongly linked to major cardiovascular events than BP measured at conventional sites like the brachial artery [16]. As a superficial vessel, the carotid artery is also easily accessible for imaging devices that measure PWV, making it convenient for PWV-based BP prediction.

## Methodology

**Fig. 1:**
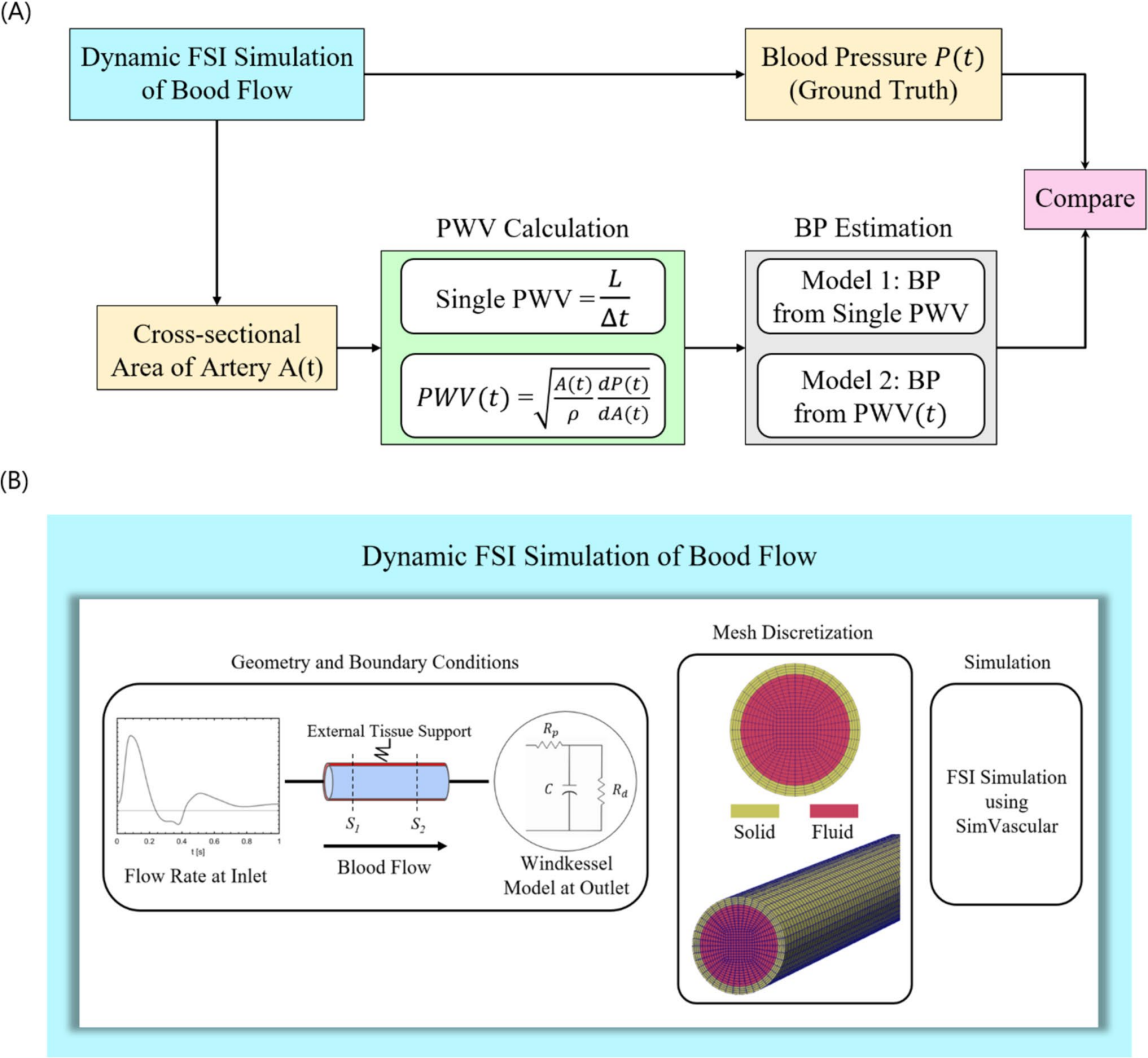
Workflow to assess the impact of using single PWV versus time-varying $PWV(t)$ on the accuracy of predicting ground truth blood pressure $P(t)$. **A** Overall study workflow. **B** Expanded view of FSI simulation

The methodology and workflow in this study are illustrated in Fig. 1. As shown in Fig. 1A, from dynamic FSI simulations, we extract time-dependent cross-sectional averaged BP $P(t)$ and inner cross-sectional area $A(t)$ of the artery. PWV is calculated by two methods based on the waveform $A(t)$, which are evaluated against the ground-truth $P(t)$. Figure 1B shows details of the FSI model, where we apply a physiological flowrate profile at the inlet, a three-element Windkessel model [37] at the outlet to generate physiological BP profiles, and external tissue support on the arterial wall. In the following sections, we elaborate more on the FSI simulations setup, the PWV calculation methods, and the BP estimation methods.

### FSI Simulation of the Artery

In this section, we describe the FSI simulation setup. The FSI simulations act as our test bed to generate physiological BP, $A(t)$, and PWV. The FSI simulations capture the two-way coupled interactions between the flow field and the vessel wall, where the velocity and pressure affect the vessel wall, and vice-versa. We use svFSI [38], a module of the open-source SimVascular [39] software to perform the simulations. There are three major steps involved in performing a FSI simulation (i) Creating the artery geometry and providing its material properties (ii) Selecting appropriate boundary conditions to describe the flow field and structural response of the geometry (iii) Computational setup of the FSI simulation. We briefly elaborate further in the following sections.

#### Geometry and Material Modeling

Both radial artery and common carotid arteries are modeled as cylindrical tubes with physiological mechanical properties. The geometrical values for the arterial length *L*, inner diameter ${D}_{0}$, and wall thickness *h* are chosen based on the literature [37, 40–43] and are provided in Table 1. Since non-Newtonian effect is minimal in large arteries, blood is modeled as a Newtonian fluid at a viscosity of 0.004 $\text{Pa s}$. The densities of blood and the artery are assumed to be $1060 \, \text{kg}/{\text{m}}^{3}$ and $1000 \, \text{kg}/{\text{m}}^{3}$, respectively [37].

**Table 1:** Geometrical and artery wall material properties

Artery		Radial artery	Common carotid artery
Inner diameter	${D}_{0}$ [mm]	2.064 [43]	6.4 [46]
Wall thickness	*h* [mm]	0.2 [40]	0.33 [41]
Artery length [37]	*L* [mm]	110	110
Poisson ratio [42]	*ν*	0.45	0.45
HGO material parameters [45]	${c}_{0}$ [kPa]	88.8	74.1
	${k}_{1}$ [kPa]	0.0505	55.6
	${k}_{2}$	50.7	11.2

To account for the nonlinear arterial wall response to BP, the artery wall is represented using the Holzapfel–Gasser–Ogden (HGO) constitutive model [36] in svFSI [38, 44]. This model is alternatively known as Gasser–Ogden–Holzapfel (GOH) model and is a generalization of the original HGO model [32]. More details of the constitutive modeling in svFSI are provided in [36, 44]. Briefly, the HGO model captures the stiffening response of the arterial wall to BP due to collagen fiber recruitment in a physiological pressure range, (*e.g.,* 30 mmHg to 150 mmHg), and the elastin fiber response at a low-pressure range (*e.g.,* 0 mmHg to 30 mmHg). The elastin fiber response is represented using a Neo-Hookean material with a shear modulus ${c}_{0}$. The collagen fiber response is represented using a stress-like stiffness parameter ${k}_{1}$, and a dimensionless parameter ${k}_{2}$. Additionally, the collagen fibers are embedded at a fiber angle with respect to the arterial circumference. For simplicity, we set the fiber orientation to 0 following ref. [45]. The values of material properties are provided in Table 1.

#### Boundary Conditions

**Fig. 2:**
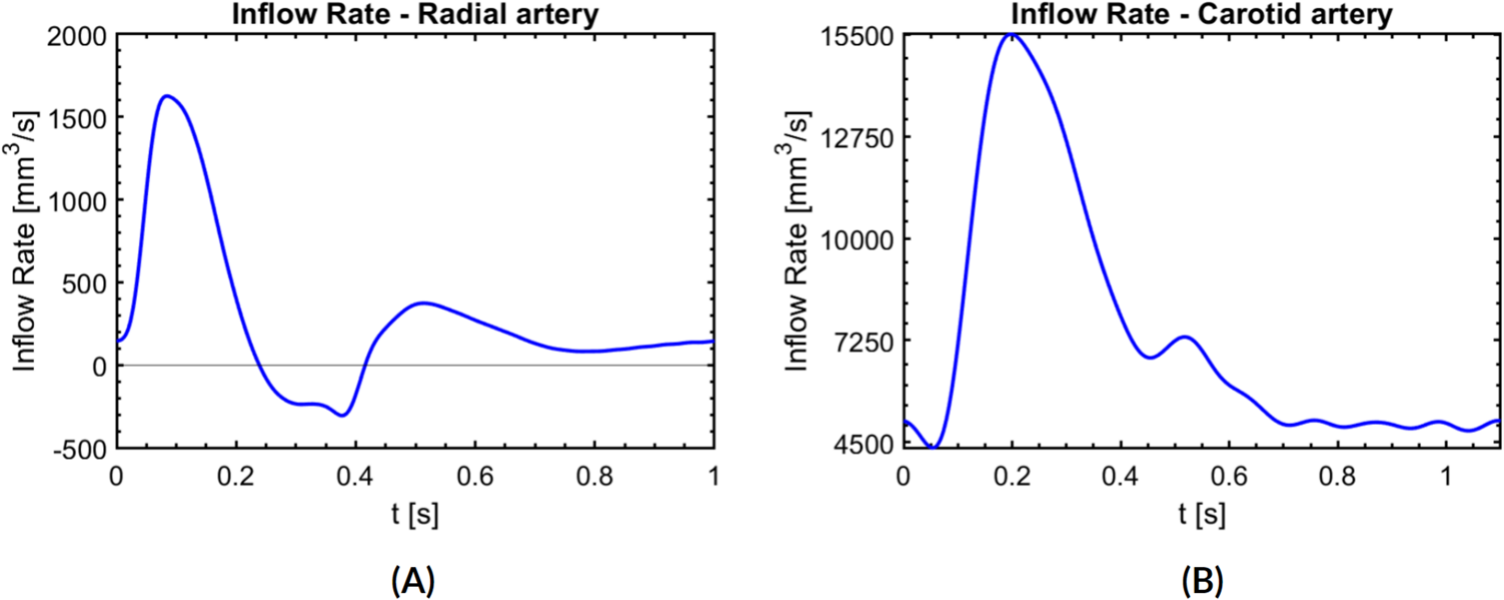
Pulsatile flow rate as inlet boundary condition. **A** Radial artery. **B** Common carotid artery

The boundary conditions for the FSI simulations are defined at the flow inlet, flow outlet and outside the arterial wall. At the inlet of the artery, we apply a physiological flow rate $Q(t)$. The inflow waveforms were obtained from the literature for the radial artery [47] and common carotid artery [48] and are shown in the Fig. 2.

At the outlet of the artery, we apply a simple three-element Windkessel model—a lumped-parameter representation of the truncated distal arterial network. It simplifies the entire distal arterial system into three elements without explicitly considering the detailed structure of the arterial tree. The Windkessel model captures the *effective impedance*, i.e. the total opposition to the pulsatile blood flow in the arterial system, which combines both resistive (resistance) and reactive (compliance) effects, as well as wave propagation properties. As illustrated in Fig. 1B, our Windkessel model consists of:

1. A capacitor $C$ to model the vascular compliance [49]. This is the ability of the arterial walls to expand and contract in response to changes in pressure.
2. A distal resistance ${R}_{d}$ to represent the resistance of the small peripheral vessels [49]. This is the opposition to blood flow due to friction within the blood vessels, primarily contributed by the smaller arteries and arterioles.
3. A proximal resistance ${R}_{p}$ (also known as arterial characteristic impedance ${Z}_{c}$) to represent the wave travel aspects of the arterial system [49]. As blood moves through arteries, pressure waves travel along the vessel walls and the fluid medium. The propagation of these waves and their interactions with vessel characteristics also contribute to the overall impedance of the system.

The Windkessel parameters were tuned over multiple simulations until a target diastolic BP and systolic BP for the physiological BP waveform are obtained [50]. We specified physiological pressure ranges of 65–85 mmHg for diastolic BP, and 110–130 mmHg for systolic BP. An initial estimate for the total resistance ${R}_{T}$ was specified as the ratio of the desired mean pressure to mean inlet flow rate [37], and an initial compliance estimate as the ratio of the time constant during exponential fall in diastole to ${R}_{T}$ [37]. We tuned ${R}_{p}$ to match the arterial characteristic impedance $\rho c/A$ of the upstream domain [37], where $\rho$ is the fluid density, $c$ is the diastolic PWV, and $A$ is the diastolic area recorded 36 mm from the inlet. The diastolic PWV was calculated at minimal pressure in the cardiac cycle using the Bramwell–Hill equation (Sect. "[Sec Sec9]"). ${R}_{d}$ was then obtained as ${R}_{T}-{R}_{p}$. The final parameters of the Windkessel model used for FSI simulations are given in Table 2. The BP waveform extracted from the simulations (*i.e.*, the simulated pressure) serves as our ground truth for the investigation of PWV-based BP estimation models.

**Table 2:** Windkessel model parameters (applied at the outlet), tuned to produce physiological pressure waveforms

Artery	Resistance 1 $\times {10}^{9}$ [$\text{Pa s }{\text{m}}^{-3}$]	Resistance 2 $\times {10}^{9}$ [$\text{Pa s }{\text{m}}^{-3}$]	Compliance $\times {10}^{-9}$ [${\text{m}}^{3}\text{ P}{\text{a}}^{-1}$]
Radial artery	2.26668	37.3104	0.032595
Carotid artery	0.2120	1.6384	0.2954

Finally, for the arterial wall boundary conditions, both the ends of the artery are assumed to have zero displacement. To mimic external tissue support, we use a Robin-type boundary condition at the outer arterial wall. The tissue stiffness parameter ${k}_{s}=1\times {10}^{6}\frac{Ns}{{m}^{3}}$ was chosen from an available literature range (${k}_{s}=0$ to ${k}_{s}=1\times {10}^{8}\frac{Ns}{{m}^{3}}$) [50, 51].

#### Meshing and FSI Solver

The artery is modeled as a cylindrical tube in Gmsh 4.11.0 [52] using geometrical values in Table 1. We then create a hexahedral mesh using Gmsh 4.11.0 [52] as shown in Fig. 1B. The nodes of the fluid and solid domains are made to coincide with one another at the fluid-solid interface to satisfy the conservation of mass and momentum.

**Fig. 3:**
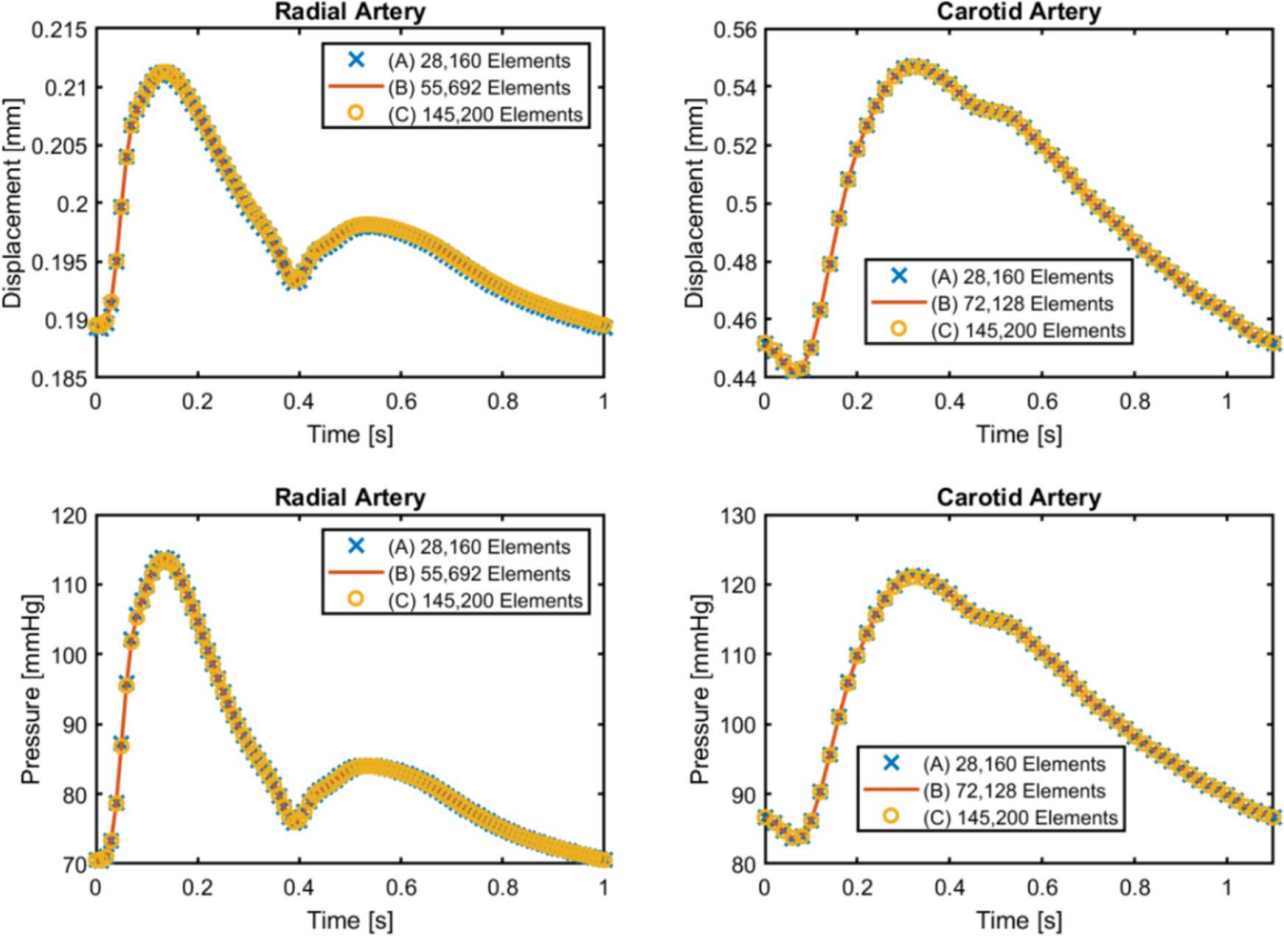
Mesh sensitivity analysis using maximum wall displacement and cross-section averaged pressure tracked over a cardiac cycle. The differences among the three refinement levels are less than 1%, achieving mesh independence

To ensure accuracy of the results, a mesh sensitivity analysis is performed at three mesh refinement levels for each arterial model. For the radial artery, the mesh consists of 28,160 (Level A; 17,600 in fluid domain and 10,560 in solid domain), 55,692 (Level B; 39,780 in fluid domain and 15,912 in solid domain), and 145,200 (Level C; 110,000 fluid domain and 35,200 solid domain) elements. For the common carotid artery, Level B has 72,128 elements (51,520 in fluid domain and 20,608 in solid domain), while its Levels A and C have the same numbers as for the radial artery. For all these mesh refinement levels, the maximum displacement of the wall and cross-section averaged pressure at a slice are tracked over a cardiac cycle, as shown in Fig. 3. For both arteries, the tracked results differ by less than 1% at these at three refinement levels, indicating that mesh independence is achieved. For maximum accuracy, we present results from Level C in this study.

FSI simulations are performed using svFSI [38], a finite element Multiphysics solver from SimVascular [39]. The governing equations of the fluid and solid domains are based on the Arbitrary-Lagrangian Eulerian (ALE) formulation described in [28], which are discretized and simultaneously solved as a strongly coupled linear system following a monolithic approach using the finite element method in svFSI. The GMRES linear iterative solver is used, with a tolerance of ${10}^{-5}$. Further details about the numerical formulation in svFSI can be found in the study by Bäumler et al. [50]. The dynamic simulations are performed over multiple cardiac cycles. To ensure cycle-to-cycle repeatability, Windkessel model requires at least 6–7 cardiac cycles. We run 9 cardiac cycles for the carotid artery and 13 cardiac cycles for the radial artery, both with a temporal resolution 1 $\text{ms}$. For each artery, we extract $P(t)$ and $A(t)$ from the last cardiac cycle and use these quantities for the calculation of PWV and BP estimation, as described in the following sections. Please note that the inner arterial area $A$ is calculated as $\frac{\pi {D}^{2}}{4}$, where $D$ is the sum of blood pressure-free diameter ${D}_{0}$​ (Table 1) and the radial displacement of two nodes at opposite ends of the mesh in a planar slice.

### PWV Calculation

#### Single PWV Calculation

In sensing devices, a PWV is generally estimated using

1 $$PWV= \frac{L}{\Delta t},$$

where $L$ is a fixed distance along the artery and $\Delta t$ is the time delay of a physiological pulse waveform, for example, the inner area waveform $A(t)$ that sensors detected at two locations along the artery separated by $L$. Traditionally, the time delay $\Delta t$ is tracked for a single point on the waveform, typically around the minimum diastolic point just before the systolic rise of the waveform [3, 53, 54]. Hence, Eq. 1 essentially represents a PWV at the diastolic phase corresponding to diastolic BP [12, 34], which can be referred to as diastolic PWV or $PW{V}_{d}$.

The single PWV-based BP estimation approach uses $PW{V}_{d}$. To implement the calculation of $PW{V}_{d}$, we use the popular intersecting tangents method [54], where a fiducial point defined by the intersection of a line passing through the minimum and a line tangent to the maximal first derivative of the waveform is tracked. The waveform we track is the inner area $A(t)$ waveform extracted from the FSI simulation, as the $A(t$) waveform can conveniently be obtained through noninvasive methods such as ultrasound [14, 20, 26, 33].

#### Time-Varying ${\varvec{P}}{\varvec{W}}{\varvec{V}}({\varvec{t}})$ Calculation

For BP estimation based on time-varying PWV, we need to obtain $PWV(t)$ from FSI. The Bramwell–Hill equation [55] expresses $PWV(t)$ as a function of $BP(t)$ and $A(t$). But in reality, $BP(t)$ is what we are trying to predict. Hence, we need another method to calculate $PWV(t)$, and then use the $PWV(t)$ to predict $BP(t)$, to be discussed in Sect. “[Sec Sec12]”. Nevertheless, since Bramwell–Hill equation is the foundation for the BP prediction models, it is presented here. Derived from the mass and momentum equations for a deformable fluid element [35] in a long, cylindrical tube containing an inviscid fluid [8, 55], the Bramwell–Hill equation is presented as:

2 $$PWV\left(t\right)= \sqrt{\frac{A\left(t\right)}{\rho }\frac{dP\left(t\right)}{dA\left(t\right)}},$$

where $\rho$ represents the fluid density, $dP$ represents the change in pressure and $dA$ represents the change in vessel area. $dP/dA$ is essentially the stiffness of the vessel or inverse of its compliance.

Equation 2 is only a theoretical formula. In practice, PWV is still measured using Eq. 1 through the time delay of an entire waveform or a specific point on a waveform and currently there is no method to directly measure a time-dependent $PWV(t)$. To represent the effect of a time-dependent $PWV(t)$, we hypothesize a positive correlation between the instantaneous $PWV(t)$ and $A\left(t\right).$ This is reasonable because a pressure increase results in an expansion and stiffening of the artery simultaneously, i.e., both $A(t)$ and $PWV(t)$ increase concurrently. As a first permutation of this hypothesis, we assume that $PWV(t)$ scales linearly with $A(t)$. This assumption will be tested using our FSI simulation results (see Sect. “[Sec Sec16]”). A linear scaling relation between $PWV(t)$ and $A(t)$ using $PW{V}_{d}$, $PW{V}_{s}$, ${A}_{d}$, ${A}_{s}$, where subscripts $d$ and $s$ represents diastolic and systolic, respectively, can be defined as

3 $$PWV\left(t\right)={PWV}_{d}+m\left(A\left(t\right)-{A}_{d}\right),$$

where $m=(PW{V}_{s}-PW{V}_{d})/({A}_{s}-{A}_{d})$. Equation 3 can be re-written as

4 $$PWV\left(t\right)={C}_{1}+mA\left(t\right),$$

where $${C}_{1}=PW{V}_{d}-m{A}_{d}$$.

The diastolic PWV ($PW{V}_{d}$) follows the method described above. Currently, we are not aware of a good method to practically and reliably estimate $PW{V}_{s}$ from the time delay of a waveform. The difficulty of calculating $PW{V}_{s}$ is that wave reflections at vessel split locations affect the systolic peak [12, 34]. Therefore, for $PW{V}_{s}$, we directly use the maximum PWV corresponding to the peak systole from the theoretical $PWV(t)$ in Eq. 2. To numerically calculate the derivative $dP(t)/dA(t)$ in Eq. 2, the gradient function is used in MATLAB [56]. The robust least-squares regression method in MATLAB [56] is used to smooth any sharp over/undershoots that arise from numerically calculating the gradient.

### Blood Pressure Estimation Models from PWV

After calculating both the single PWV ($PW{V}_{d}$) and time-varying $PWV(t)$ from FSI simulated flow field, we can proceed to evaluating their ability of using these two strategies to predict BP accurately. To that end, we rearrange the Bramwell–Hill equation (Eq. 2) to express BP in terms of PWV:

5 $$P\left(t\right)={P}_{0}+\rho \underset{{A}_{0}}{\overset{A}{\int }}PW{V}^{2}(t)\frac{dA(t)}{A(t)},$$

where, ${A}_{0}$ is the inner arterial area measured at a pressure ${P}_{0}$. For in vitro experiments and in vivo, ${P}_{0}$ is the calibration pressure, typically measured as a diastolic BP (${P}_{d}$) obtained using routine calibration from a cuff-based device such as a sphygmomanometer [19]. For our study, we use ${P}_{0}={P}_{d}$ and ${A}_{0}={A}_{d}$ extracted at the same diastolic time point from the simulation.

#### Model 1: PWV-Based BP Estimation Using Single PWV

The common approach to handling the integration in Eq. 5 is to assume $PWV\left(t\right)=constant$ during the cardiac cycle (*e.g.,* [14, 15, 18, 19, 23–26, 57]). Under this assumption, Eq. 5 becomes

6 $$P\left(t\right)={P}_{0}+\rho PW{V}^{2}{\text{ln}}\left(\frac{A(t)}{{A}_{0}}\right),$$

where the constant $PWV$ is usually taken as $PW{V}_{d}$:

7 $$P\left(t\right)={P}_{0}+\rho {PW{V}_{d}}^{2}{\text{ln}}\left(\frac{A(t)}{{A}_{0}}\right)$$

Equation 7 is our BP-prediction Model 1 (M1) to be tested.

#### Model 2: PWV-Based BP Estimation Using Time-Varying ${\varvec{P}}{\varvec{W}}{\varvec{V}}({\varvec{t}})$

To close the integration in Eq. 5 without assuming $PWV=constant$, we plug the scaled $PWV(t)$ given in Eq. 4 into Eq. 5 and obtain

8 $$P\left( t \right) = P_{0} + \rho \left( {C_{1}^{2} {\text{ln}}\left\lfloor {\frac{{A\left( t \right)}}{{A_{0} }}} \right\rfloor + m^{2} \left\lfloor {\frac{{A^{2} \left( t \right) - A_{0}^{2} }}{2}} \right\rfloor + 2C_{1} m\left[ {A\left( t \right) - A_{0} } \right]} \right),$$

where

$$m=(PW{V}_{s}-PW{V}_{d})/({A}_{s}-{A}_{d})$$ and $${C}_{1}=PW{V}_{d}-m{A}_{d}$$.

Equation 8 is our BP-prediction Model 2 (M2) to be tested.

### How We Test the BP Prediction Models

Using data extracted from FSI simulations, we test BP prediction models M1 and M2, which are based on a single PWV and time-varying $PWV(t)$, respectively. To obtain $PW{V}_{d}$, we take the time delay $\Delta t$ of the $A(t)$ waveform at two planes, 36 mm and 96 mm from the inlet, respectively, marked as ${S}_{1}$ and ${S}_{2}$ in Fig. 1B. Combining $PW{V}_{d}$ with the locally measured $A(t)$, ${A}_{d}$, and ${P}_{0}$ at ${S}_{1}$ and ${S}_{2}$, we perform the local BP predictions from M1 based on Eq. 7. To obtain $PW{V}_{s}$ at ${S}_{1}$ and ${S}_{2}$, we take the maximum value on the theoretical $PWV(t)$ calculated using the Bramwell–Hill equation (Eq. 2). Combining the single $PW{V}_{d}$ and the locally measured $PW{V}_{s}$ with the locally measured $A(t)$, ${A}_{d}$, and ${P}_{0}$ at ${S}_{1}$ and ${S}_{2}$, we perform the local BP predictions from M2 based on Eq. 8.

## Results

In this section we first present the ground truth $P(t)$ and $A(t)$, along with the theoretical $PWV(t)$, followed by BP prediction results using a single PWV (M1) and using a time-varying $PWV(t)$ (M2). Please note that since we calibrate both prediction models using the ground truth ${P}_{0}$ (diastolic BP), there will be no observed error in diastolic BP during the cardiac cycle when ${P}_{0}$ matches the diastolic BP.

### Ground Truth Pressure ${\varvec{P}}({\varvec{t}})$, Inner Arterial Area ${\varvec{A}}({\varvec{t}})$, and Pulse Wave Velocity ${\varvec{P}}{\varvec{W}}{\varvec{V}}({\varvec{t}})$

**Fig. 4:**
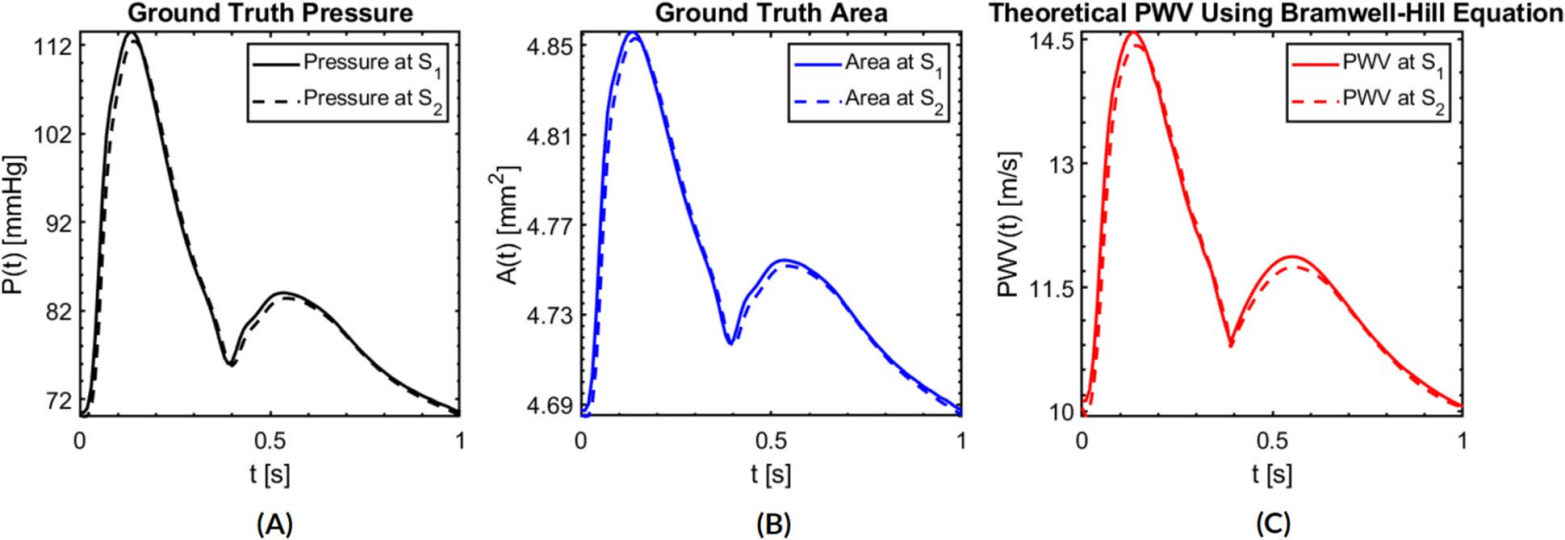
The ground truth quantities in a cardiac cycle at planes S_1_ and S_2_ of the radial artery. **A** The ground truth *P*(*t*). **B** The waveform “signal” *A*(*t*) required for PWV-based BP estimation models. **C** Theoretical $PWV(t)$ based on the Bramwell–Hill equation

**Fig. 5:**
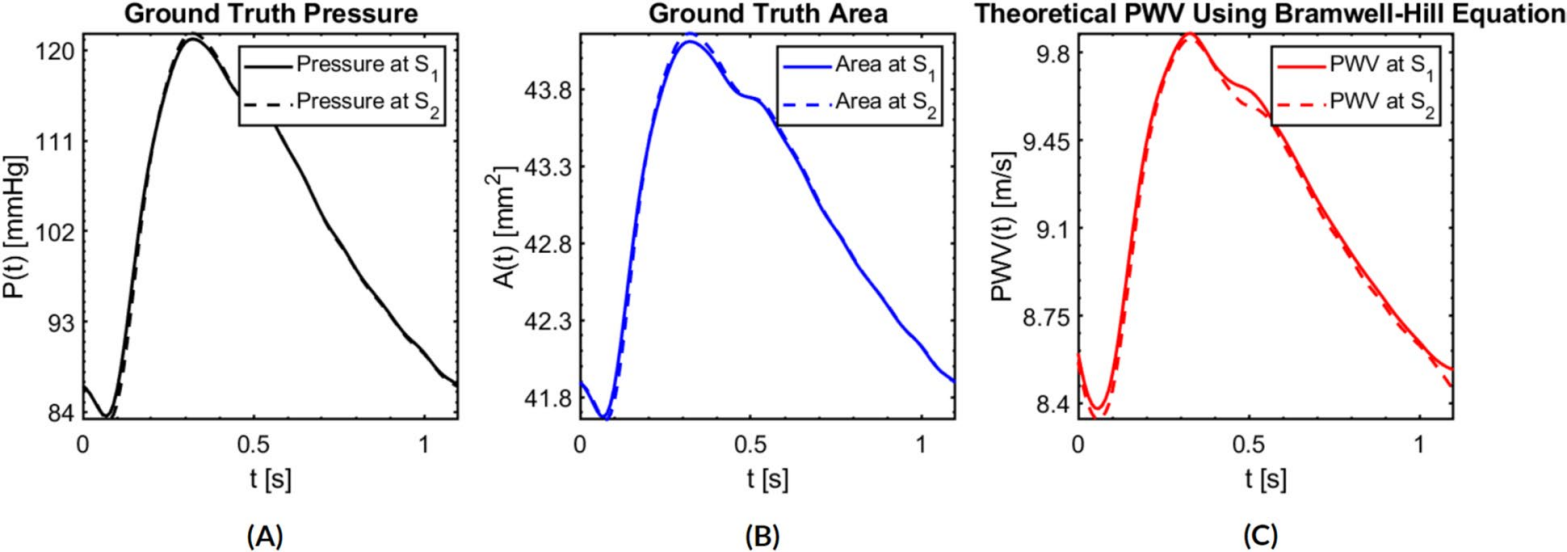
The ground truth quantities in a cardiac cycle at planes S_1_ and S_2_ of the common carotid artery. **A** The ground truth pressure *P*(*t*). **B** The waveform “signal” *A*(*t*) required for PWV-based BP estimation models. **C** Theoretical $PWV(t)$ based on the Bramwell–Hill equation

Figures 4 and 5 show the FSI output of $P(t)$ and $A(t)$ extracted at the two axial locations ${S}_{1}$ and ${S}_{2}$ along with the theoretical $PWV(t)$ calculated from the Bramwell–Hill equation (Eq. 2) in one cardiac cycle, respectively for the radial and common carotid arteries. These waveforms can be viewed as the ground truths in our numerical testing. Specifically, $P(t)$ provides the ground truth for BP model predictions, *A*(*t*) is the waveform “signal” fed to the PWV-based BP estimation models, and the theoretical $PWV(t)$ provides physical insight into PWV behavior. Evidently, the temporal variations of vessel area *A*(*t*) and $PWV(t)$ qualitatively mirror the temporal variation of pressure $P(t)$. The PWV experiences as much as a 47.0 % variation between the minimum (9.93 m/s) and maximum (14.59 m/s) for the radial artery, and an 18.5 % variation between the minimum (8.33 m/s) and maximum (9.87 m/s) for the common carotid artery.

### Justification of Linear Scaling of ${\varvec{P}}{\varvec{W}}{\varvec{V}}({\varvec{t}})$ to ${\varvec{A}}({\varvec{t}})$

**Fig. 6:**
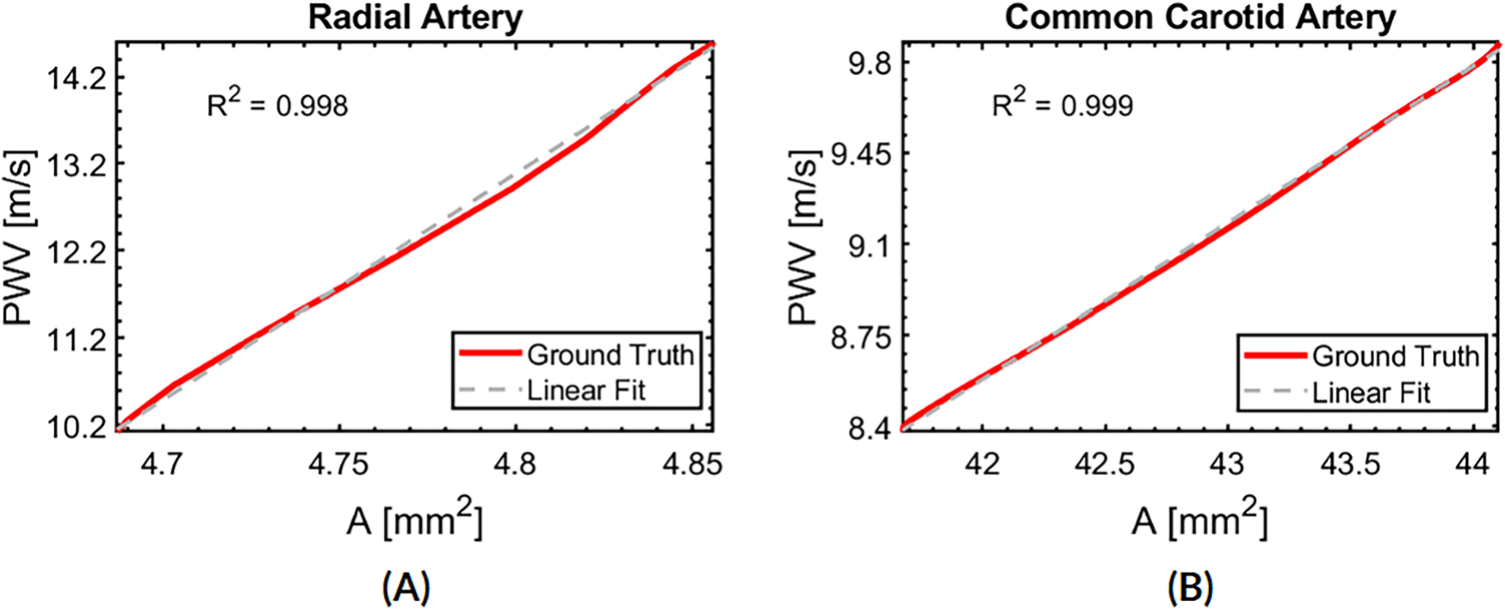
Linear relationship between $A(t)$ and $PWV$. **A** Radial artery. **B** Common carotid artery

To obtain a time-dependent PWV, in Sect. "[Sec Sec9]" we hypothesized a linear relationship between $PWV(t)$ and $A(t)$. Here we test this hypothesis using the ground truth FSI results shown in (B) and (C) of Figs. 4 and 5. We plot instantaneous $PWV$ vs $A$ during the systolic upstroke at axial location ${S}_{1}$ in Fig. 6. Evidently, the $PWV$ and $A$ have an excellent linear relationship, with ${R}^{2}$ values > 0.998. This result justifies our proposed linear scaling formula between $PWV(t)$ and $A(t)$ in Eq. 4.

### Model Predictions of Blood Pressure

**Fig. 7:**
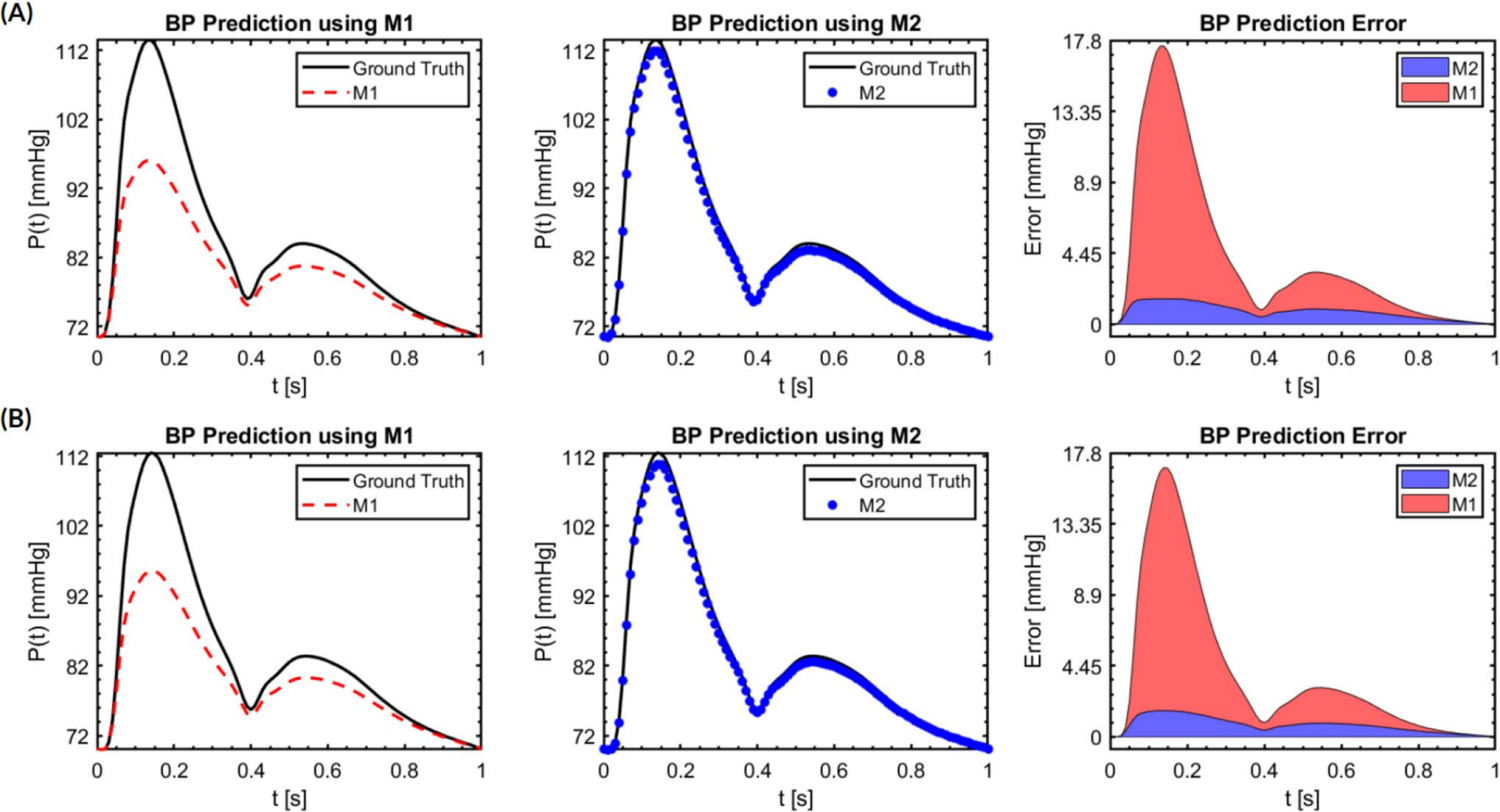
BP prediction results and their errors in a cardiac cycle using M1 and M2 compared against the ground truth at the two evaluation planes in the radial artery. **A** Location ${S}_{1}$. **B** Location ${S}_{2}$

**Fig. 8:**
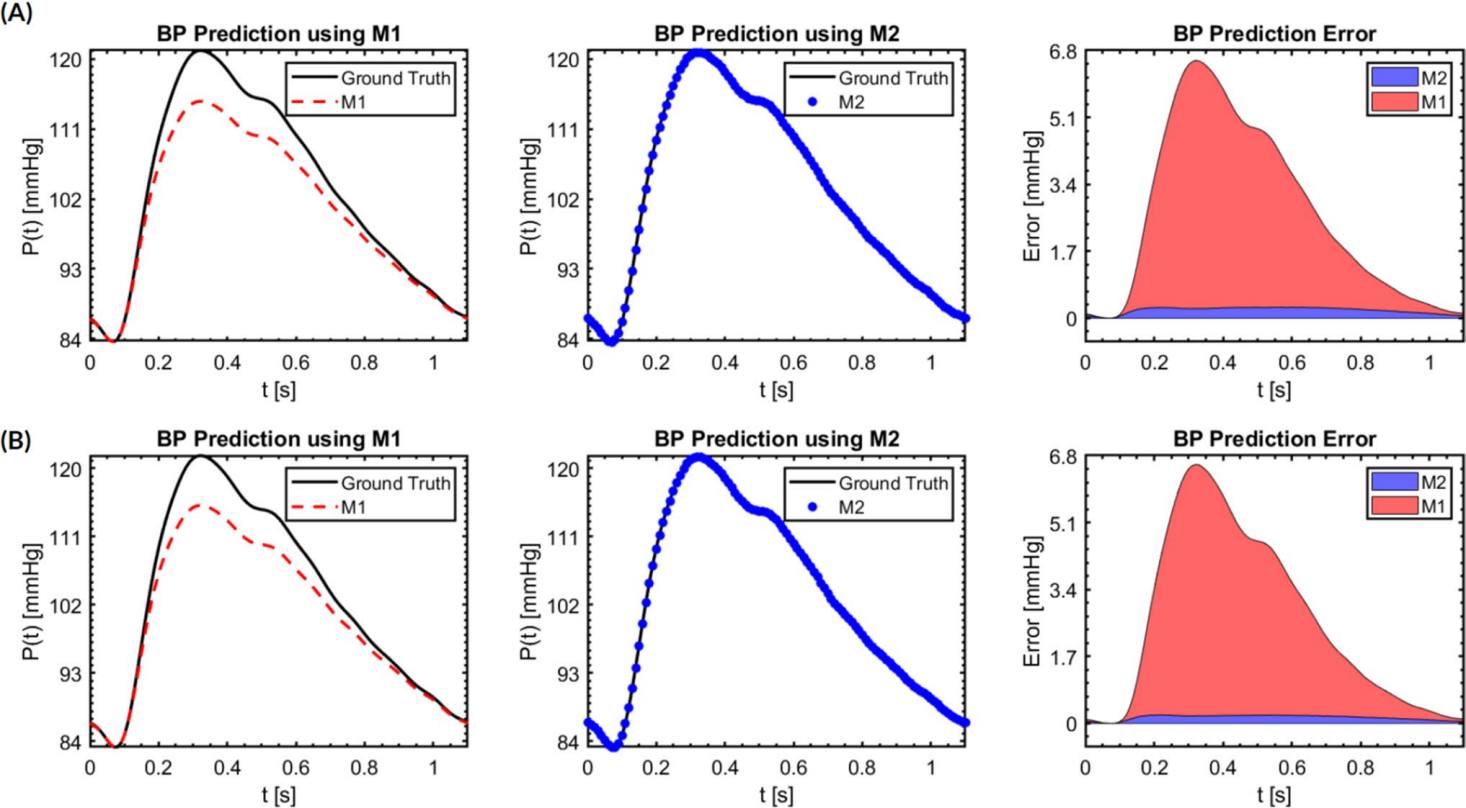
BP prediction results and their errors in a cardiac cycle using M1 and M2 compared against the ground truth at the two evaluation planes in the common carotid artery. **A** Location ${S}_{1}$. **B** Location ${S}_{2}$

Figures 7 and 8 illustrate BP prediction results by the two models, M1 (red dashed line) and M2 (blue dots), compared against the ground truth (solid line), at locations ${S}_{1}$ and ${S}_{2}$ in the radial artery and common carotid artery, respectively. In the radial artery (Fig. 7), the single PWV model (M1) exhibits significant errors in BP prediction, particularly at peaks, with the largest errors occurring at peak systole: 17.44 mmHg at location ${S}_{1}$ and 16.88 mmHg at ${S}_{2}$. On the contrary, the time-varying PWV model (M2) closely replicates the ground truth BP waveform at both ${S}_{1}$ and ${S}_{2}$, with peak systole errors of 1.59 mmHg and 1.63 mmHg, respectively. Similarly, in the common carotid artery (Fig. 8), the single PWV model (M1) shows considerable prediction errors at peaks, with the largest errors being 6.54 mmHg at location ${S}_{1}$ and 6.57 mmHg at ${S}_{2}$. The time-varying PWV model (M2), however, accurately reproduces the entire ground truth BP waveform at both locations, with errors of only 0.24 mmHg and 0.18 mmHg at peak systole, respectively. By comparing the prediction errors from M1 and M2 side by side, Table 3 summarizes the main findings of this study, *i.e.*, the drastic improvement of BP prediction using time-varying PWV, reducing prediction errors by 90.6% and 96.8% in the radial and carotid artery, respectively.

**Table 3:** Comparison of BP prediction errors using Model 1 and Model 2, showing drastic error reduction by Model 2

	Prediction errors for systolic BP			
	Model 1 (Eq. 7) Based on $PW{V}_{d}$ (mmHg)	Model 2 (Eq. 8) Based on $PW{V}_{d}$ and $PW{V}_{s}$ (mmHg)	Error reduction by Model 2 (%)	Average error reduction by Model 2 (%)
Radial artery				
Plane ${S}_{1}$	17.44	1.59	90.9	90.6
Plane ${S}_{2}$	16.88	1.63	90.3	
Carotid artery				
Plane ${S}_{1}$	6.54	0.24	96.3	96.8
Plane ${S}_{2}$	6.57	0.18	97.3	

Note that the prediction error for diastolic BP is zero, since we took the ground-truth diastolic BP as the calibration BP (${P}_{0}$)

## Discussion

This study highlights the importance of temporal variations of PWV in BP prediction. Our motivation stemmed from the observation that almost all the existing PWV-based BP prediction models (*e.g*., [3, 6, 14, 15, 18–21, 23–26]) employ a single, constant PWV, and they often suffer from persistent inaccuracies [3]. The temporal variation of PWV has only been occasionally mentioned in the literature. Nabeel et al. [34] described an empirical relation of $PWV(t)$ as a function of BP and inner arterial diameter within the cardiac cycle. Meinders and Hoeks [33] depicted the variation of $PWV$ using a curve-fitted relation between BP and area recorded from a cardiac cycle. These approaches require the knowledge of the time-dependent BP to estimate the $PWV(t)$, but for BP monitoring devices, the BP itself is the unknown parameter to be predicted in real-time.

In general, current PWV-based cuffless wearable devices utilize two types of BP estimation models. The first type of BP models are derived from the Moens–Korteweg equation [3, 6, 27], which assumes linear elasticity in the arterial wall [28]. The second type of BP models, like in Ma et al. [8], assume a constant PWV while not explicitly stemming from the Moens–Korteweg equation. Despite starting with a nonlinear arterial wall assumption in their derivations, these authors simplified their BP formula to depend on a single, constant PWV [8]. We have recently demonstrated that the linear arterial wall assumption leads to a constant PWV throughout the cardiac cycle, and a constant PWV implies the linear arterial wall assumption [28]. Therefore, both types of BP models are essentially similar in their underlying assumption of linear elasticity, which diverges from the behavior of real arteries.

The linear elastic material assumption is appropriate only for thin-walled vessels undergoing negligible deformations [28]. In reality, arteries exhibit hyperelastic behavior: the stiffness of arteries increases with blood pressure due to increasing engagement of collagen fibers [29]. Moreover, arteries typically have wall thickness > 10% of the radius [40] and can undergo large, nonlinear deformations of up to 30% of the radius [32] in vivo with a changing compliance. Bantwal et al. [28] have demonstrated that the nonlinear arterial response to BP can result in 20% to 50% variation of PWV in a cardiac cycle, consistent with in vivo results in the literature [11, 33]. The significant temporal variation of $PWV(t)$ calls into question whether a single PWV is sufficient to reliably predict BP.

To assess the impact of using a single PWV on BP prediction accuracy, we compared two BP estimation models: M1, a commonly used method (*e.g.*, [14, 15, 18, 19, 23, 25]) based on a single PWV, and M2, a novel model that incorporates time-varying PWV. We showed that M1 *underpredicts* the systolic BP by up to 17.44 mmHg in the radial artery and 6.47 mmHg in the carotid artery, while the M2 model reduces these errors by > 90% to 1.63 mmHg and 0.24 mmHg, respectively (see Figs. 7, 8 and Table 3). Our current study does not show errors in predicted diastolic BP due to using the ground-truth diastolic BP for calibration (${P}_{0}$). However, the predicted diastolic BP values in practical measurement devices are likely subjected to similar errors as systolic BP, since the BP estimation models often contain different coefficients trained or regressed separately for diastolic BP and systolic BP from human data [3].

Our study has demonstrated that relying on a single PWV, which implies the linear elastic assumption for the artery, can lead to severe underpredictions of BP. This finding has significant clinical implications: underestimation of BP can mask hypertension, a major risk factor for cardiovascular diseases and diabetes [1, 58]. Not being timely diagnosed and treated, hypertension can worsen the progression of cardiovascular risks and increase the likelihood of adverse events such as stroke, myocardial infarction, heart failure, and sudden cardiac death [58, 59]. Therefore, it is crucial to account for the temporal PWV variations for PWV-based noncontact BP monitoring devices. Assuming a constant PWV may only be reasonable if PWV variations are small, but this is a dangerous *a priori* assumption. Figures 4C and 5C show that PWV varies by 47% in the radial artery case and 18% in the CCA case, and consequently the BP prediction error from the single-PWV model M1 is larger in the radial artery than in the CCA case. However, large PWV variations have been reported in CCA. In an ex vivo study, Bramwell and Hill [55] showed that PWV in CCA could vary as much as 72% (4.81–8.3 m/s over a pressure range of $\approx$ 32 mmHg). Therefore, in general, the single-PWV based BP estimation cannot be relied upon regardless of the artery.

Unfortunately, the implications of the linear assumption have rarely been recognized in the field of cuffless BP monitoring technology development. Emerging techniques are generally focused on creating and improving sensing devices [3, 5] (often based on ultrasound [14, 24, 60] or piezoelectric principles [20]). Errors in BP prediction are commonly attributed to sensor noises, insufficient recalibrations, and hardware limitations [3]. Our study sheds light on the importance of the assumed arterial mechanics underlying any BP prediction approaches, suggesting that accounting for the nonlinear arterial behavior (*i.e.,* the time-varying PWV) is critical to achieving high BP prediction accuracy.

To overcome the limitation of single-PWV models, we have proposed a novel BP prediction model (M2, Eq. 8) that accounts for the effects of $PWV(t)$ or the nonlinear arterial behavior, achieving greater than 90% reduction in BP prediction error compared to a single-PWV model. This new model, M2, incorporates the effect of a time-varying PWV but does not require the direct measurement of $PWV(t)$. It incorporates a simple linear scaling of the time-varying cross-sectional area $A(t)$, which can be dynamically measured using existing sensors such as ultrasound [5], in combination with two PWV values, $PW{V}_{d}$ and $PW{V}_{s}$. The linear relationship between $PWV(t)$ and $A(t)$ is confirmed by our FSI results (Fig. 6), lending credence to our representation of $PWV\left(t\right)$ using $A(t)$. As long as the two PWV values, $PW{V}_{d}$ and $PW{V}_{s},$ are measured within a cardiac cycle, the M2 algorithm in Eq. 8 can be easily implemented in consumer-grade devices without the need for intensive real-time $PWV(t)$ computation. Compared to the single-PWV-based BP model, our novel BP model significantly improves BP prediction accuracy without requiring dynamic PWV data processing in real-time.

The current challenge, however, is obtaining an accurate measurement of $PW{V}_{s}$. Measuring $PW{V}_{s}$ requires tracking the time delay at the systolic peak between two locations, but this is challenging because the wave shape is often distorted by wave reflections, making it difficult to accurately identify the systolic peak on the combined waveform [12, 34, 61]. Wave reflection happens whenever a propagating wave encounters an impedance mismatch, which arises when the characteristics of the arteries vary at different points along the arterial tree (e.g., arterial diameter, stiffness, and compliance). When a pressure wave encounters a region of the artery with a sudden change in characteristics like bifurcations or branching, arterial geometric tapering (gradual decrease in arterial area), elastic tapering (gradual increase in wall stiffness), or resistance vessels (arterioles) [62], some of the wave is reflected back toward the heart. This reflected wave can interfere with the incoming wave, creating phase shifts and attenuations between the pressure and flow waveforms. More importantly, the phase-shifted reflective wave is superposed onto the incoming wave, changing the shape of the $A(t)$ waveform differently at different points along the artery. This makes it challenging to identify the same systolic point on the waveform between two points to obtain an accurate $\Delta t$. Therefore, accurate measurement of $PW{V}_{s}$ has been problematic. In contrast, the $PW{V}_{d}$ uses the delay of a fiducial point near the foot, which is generally thought to be free of contamination by reflections [34].

Efforts towards obtaining $PW{V}_{ s}$ or other temporal $PWV$ estimates have so far met with limited success. Chen et al. [12] proposed a method to obtain $PW{V}_{ s}$ using the long time delay on a photoplethysmogram (PPG) wave traveling from ear to toe. The characteristic point to calculate $PW{V}_{ s}$ is specific to the ear and toe wave shapes. Since their approach is specific for the ear-to-toe pulse involving an extremely large distance, it is not suitable for wearable device implementation. Similarly, Nabeel et al. [16] attempted to get a second PWV by identifying a characteristic point that is 0.25 units upstroke of the diastolic point on a normalized PPG cycle. However, one cannot assume that this upstroke characteristic point is immune from distortion by reflections [54]. In another study, Lillie et al. [63] estimated the $PW{V}_{s}$ by adding the aorta systolic blood flow velocity onto $PW{V}_{d}$ measured at the finger. The rationale for using the aortic blood flow velocity to estimate PWV is unclear. In an ex vivo study, Manoj et al. [64] proposed a method to calculate $PW{V}_{s}$ based on the time delay of the second derivative minima (SDMin). Their rationale was that the SDMin aligns with a point proximal to the systolic point. However, such an approach will also suffer from reflections [61] and may not be reliable when applied in vivo.

While the prevalent BP prediction models in cuffless BP monitoring technologies have been based on PWV, alternative algorithms not relying on PWV have also emerged. For instance, a recent study by Jimenez et al. [65] introduced a BP prediction technique based on ultrasound measurement of radius, wall thickness, and resonant frequency. However, their reliance on thin-wall, linear assumption may limit the accuracy of this model, resulting in discrepancies of up to 20 mmHg in the CCA when compared to cuff-based measurements. Zhou et al. [66] evaluated an empirical exponential BP-area relation [33] using a conformal ultrasound patch. This approach requires calibration of a stiffness index ($\alpha$) using the systolic BP and diastolic BP along with the corresponding areas, assuming that α remains constant after a one-time calibration. However, Kawasaki et al. [30] show that $\alpha$ can vary with age and vasoactive stimuli. Given that artery stiffness is closely related to PWV, future research could explore how incorporating $PWV(t)$ could help the dynamic evaluation of $\alpha$, potentially enhancing the accuracy of the stiffness-based BP prediction model.

This study has the following limitations, which highlight areas for future research.

1. *Idealized arterial geometry*: Real arteries exhibit subject-specific anatomical variations and non-uniform mechanical wall properties. For simplicity, we assumed an idealized cylindrical arterial geometry with uniform wall material properties. While the radial artery and common carotid artery are relatively cylindrical and have less pronounced anatomical variations compared to other arteries, this assumption may not fully reflect the complexities of arterial structures. Future research should explore how geometric variations, such as artery tapering, impact the BP prediction formula.
2. *Simplified tissue model*: To manage computational costs, we used a Robin-type boundary condition to simulate uniform tissue support surrounding the cylindrical artery. However, the presence of bones and tissue would likely complicate the artery’s mechanical behavior and wave propagation, potentially affecting PWV. Further investigation is needed to understand the influence of bones and surrounding tissue on BP prediction formulas, which may require adjustments for improved accuracy.
3. *Single numerical experiment*: In this study, we used simulated pulsatile blood flow in a compliant arterial model with a fixed set of boundary conditions including inflow waveforms from the literature to investigate BP prediction models. However, physiological data are very diverse, varying widely with physiological states, such as periods of high stress, illness, exercise, or sleep. Nonetheless, changes in physiological conditions are likely to affect parameters like flow rate, distal vascular resistance, heart rate, and shapes of $P(t)$ and $A(t)$ more than the BP-area-PWV relationships such as the Bramwell–Hill equation (Eq. 2). These relationships are determined by the underlying mechanical properties of the artery wall, which are the basis for our BP prediction model. While we anticipate that our proposed BP estimation model will remain robust to physiological variations, future studies should examine how different physiological states influence BP estimation.
4. *Simple Outlet Boundary Condition*: While our three-element Windkessel model offers a useful approximation of distal arterial hemodynamics by modeling effective impedance, it does not reveal the causes or sources of wave reflections. As a lumped parameter model, it limits our systematic understanding of the underlying factors contributing to wave reflections. Although wave reflections in the arterial tree have been explored in the literature [62], future studies could turn to developing more advanced boundary conditions that model the reflection phenomena within numerical simulations. Such studies would help investigate how wave reflections alter incoming waveform shapes and may motivate more research to develop practical methods to eliminate wave reflections and obtain time-dependent $PWV(t),$ such that it can be implemented in wearable devices.

Taken together, future efforts should explore expansion of the FSI model to account for the variations mentioned above, with a primary focus on accurately measuring systolic PWV. Data-driven approaches, such as machine learning could be applied to in vivo and FSI-simulated data to estimate $PW{V}_{s}$. Most importantly, BP estimation models must undergo clinical validation using large, diverse in vivo datasets.

In summary, this study demonstrates that relying on only a single PWV to predict BP can lead to significant errors. Since the arterial wall undergoes large, nonlinear deformations, PWV varies significantly in a cardiac cycle due to stiffness changes associated with BP fluctuations. Our findings show that considering $PWV(t)$, or at least $PW{V}_{s}$, is essential for accurate BP prediction, potentially cutting down more than 90% of prediction errors. Therefore, further research is needed to accurately measure PWV at the systole, which is critical for advancing noncontact BP monitoring technologies.

## References

[1] Mensah, G. A., and D. W. Brown. An overview of cardiovascular disease burden in the United States. *Health Aff. (Millwood)*. 26(1):38–48, 2007. 17211012 10.1377/hlthaff.26.1.38

[2] Kalehoff, J. P., and S. Oparil. The story of the silent killer: a history of hypertension: its discovery, diagnosis, treatment, and debates. *Curr. Hypertens. Rep.* 22(9):72, 2020. 32852612 10.1007/s11906-020-01077-7

[3] Sharma, M., et al. Cuff-less and continuous blood pressure monitoring: a methodological review. *Technologies*. 5(2):21, 2017.

[4] O’brien, E., B. Waeber, G. Parati, J. Staessen, and M. G. Myers. Blood pressure measuring devices: recommendations of the European Society of Hypertension. *BMJ*. 322(7285):531–536, 2001. 11230071 10.1136/bmj.322.7285.531 PMC1119736

[5] Mukkamala, R., G. S. Stergiou, and A. P. Avolio. Cuffless blood pressure measurement. *Annu. Rev. Biomed. Eng.* 24(1):203–230, 2022. 35363536 10.1146/annurev-bioeng-110220-014644

[6] Barvik, D., M. Cerny, M. Penhaker, and N. Noury. Noninvasive continuous blood pressure estimation from pulse transit time: a review of the calibration models. *IEEE Rev. Biomed. Eng.* 15:138–151, 2021. 10.1109/RBME.2021.3109643 34487496

[7] Quan, X., et al. Advances in non-invasive blood pressure monitoring. *Sensors*. 21(13):4273, 2021. 34206457 10.3390/s21134273 PMC8271585

[8] Ma, Y., et al. Relation between blood pressure and pulse wave velocity for human arteries. *Proc. Natl. Acad. Sci.* 115(44):11144–11149, 2018. 10.1073/pnas.1814392115. 30322935 10.1073/pnas.1814392115 PMC6217416

[9] Chen, W., T. Kobayashi, S. Ichikawa, Y. Takeuchi, and T. Togawa. Continuous estimation of systolic blood pressure using the pulse arrival time and intermittent calibration. *Med. Biol. Eng. Comput.* 38:569–574, 2000. 11094816 10.1007/BF02345755

[10] Fung, P., G. Dumont, C. Ries, C. Mott, and M. Ansermino. Continuous noninvasive blood pressure measurement by pulse transit time. In: The 26th annual International Conference of the IEEE Engineering in Medicine and Biology Society. IEEE, 2004, pp. 738–741. 10.1109/IEMBS.2004.1403264 17271783

[11] Chen, Y., C. Wen, G. Tao, M. Bi, and G. Li. Continuous and noninvasive blood pressure measurement: a novel modeling methodology of the relationship between blood pressure and pulse wave velocity. *Ann. Biomed. Eng.* 37:2222–2233, 2009. 19603270 10.1007/s10439-009-9759-1

[12] Chen, Y., C. Wen, G. Tao, and M. Bi. Continuous and noninvasive measurement of systolic and diastolic blood pressure by one mathematical model with the same model parameters and two separate pulse wave velocities. *Ann. Biomed. Eng.* 40:871–882, 2012. 22101758 10.1007/s10439-011-0467-2

[13] Liu, Z.-D., et al. Cuffless blood pressure measurement using smartwatches: a large-scale validation study. *IEEE J. Biomed. Health Inform.* 2023. 10.1109/JBHI.2023.3278168. 37204948 10.1109/JBHI.2023.3278168

[14] Seo, J., H.-S. Lee, and C. G. Sodini. Non-invasive evaluation of a carotid arterial pressure waveform using motion-tolerant ultrasound measurements during the Valsalva maneuver. *IEEE J. Biomed. Health Inform.* 25(1):163–174, 2020. 10.1109/JBHI.2020.2995344 32750903

[15] Xu, L., et al., Continuous and noninvasive measurement of arterial pulse pressure and pressure waveform using an image-free ultrasound system, ArXiv Prepr. ArXiv230517896, 2023.

[16] Nabeel, P., J. Jayaraj, K. Srinivasa, S. Mohanasankar, and M. Chenniappan. Bi-modal arterial compliance probe for calibration-free cuffless blood pressure estimation. *IEEE Trans. Biomed. Eng.* 65(11):2392–2404, 2018. 30130174 10.1109/TBME.2018.2866332

[17] Amado Rey, A. B. Estimation of blood pressure by image-free, wearable ultrasound. *Artery Res.* 30(1):3, 2024.

[18] Seabra, A. C. G., A. F. da Silva, T. Stieglitz, and A. B. Amado-Rey. In silico blood pressure models comparison. *IEEE Sens. J.* 22(23):23486–23493, 2022.

[19] Vappou, J., J. Luo, K. Okajima, M. Di Tullio, and E. Konofagou. Non-invasive measurement of local pulse pressure by pulse wave-based ultrasound manometry (PWUM). *Physiol. Meas.* 32(10):1653, 2011. 21904023 10.1088/0967-3334/32/10/012 PMC4005495

[20] Guo, C.-Y., C.-C. Chang, K.-J. Wang, and T.-L. Hsieh. Assessment of a calibration-free method of cuffless blood pressure measurement: a pilot study. *IEEE J. Transl. Eng. Health Med.* 2022. 10.1109/JTEHM.2022.3209754. 38163041 10.1109/JTEHM.2022.3209754 PMC10756135

[21] McCarthy, B., C. Vaughan, B. O’flynn, A. Mathewson, and C. Ó. Mathúna. An examination of calibration intervals required for accurately tracking blood pressure using pulse transit time algorithms. *J. Hum. Hypertens.* 27(12):744–750, 2013. 23698006 10.1038/jhh.2013.41

[22] Ding, X., and Y.-T. Zhang. Pulse transit time technique for cuffless unobtrusive blood pressure measurement: from theory to algorithm. *Biomed. Eng. Lett.* 9:37–52, 2019. 30956879 10.1007/s13534-019-00096-x PMC6431352

[23] Beulen, B. W., N. Bijnens, G. G. Koutsouridis, P. J. Brands, M. C. Rutten, and F. N. van de Vosse. Toward noninvasive blood pressure assessment in arteries by using ultrasound. *Ultrasound Med. Biol.* 37(5):788–797, 2011. 21439720 10.1016/j.ultrasmedbio.2011.01.020

[24] Xu, L., et al. Online continuous measurement of arterial pulse pressure and pressure waveform using ultrasound. *Measurement*. 220:113379, 2023.

[25] Joseph, J., P. Nabeel, M. I. Shah, and M. Sivaprakasam. Arterial compliance probe for calibration free pulse pressure measurement. In: 2016 IEEE International Symposium on Medical Measurements and Applications (MeMeA). IEEE, 2016, pp. 1–6.

[26] Seo, J., S. J. Pietrangelo, H.-S. Lee, and C. G. Sodini. Noninvasive arterial blood pressure waveform monitoring using two-element ultrasound system. *IEEE Trans. Ultrason. Ferroelectr. Freq. Control*. 62(4):776–784, 2015. 25881355 10.1109/TUFFC.2014.006904

[27] Gan, L., C. Wang, H. Liu, and L. Zhang. Cuff-less methods for blood pressure measurement. In: 2019 Chinese Automation Congress (CAC). IEEE, 2019, pp. 4926–4930.

[28] Bantwal, A. S., A. K. Bhayadia, and H. Meng. Critical role of arterial constitutive model in predicting blood pressure from pulse wave velocity. *Comput. Biol. Med.* 178:108730, 2024. 38917535 10.1016/j.compbiomed.2024.108730

[29] Fung, Y. Biomechanics: Mechanical Properties of Living Tissues. New York: Springer, 2013.

[30] Kawasaki, T., S. Sasayama, S.-I. Yagi, T. Asakawa, and T. Hirai. Non-invasive assessment of the age related changes in stiffness of major branches of the human arteries. *Cardiovasc. Res.* 21(9):678–687, 1987. 3328650 10.1093/cvr/21.9.678

[31] Hayashi, K., H. Handa, S. Nagasawa, A. Okumura, and K. Moritake. Stiffness and elastic behavior of human intracranial and extracranial arteries. *J. Biomech.* 13(2):175–184, 1980. 7364778 10.1016/0021-9290(80)90191-8

[32] Holzapfel, G. A., T. C. Gasser, and R. W. Ogden. A new constitutive framework for arterial wall mechanics and a comparative study of material models. *J. Elast. Phys. Sci. Solids*. 61:1–48, 2000.

[33] Meinders, J. M., and A. P. Hoeks. Simultaneous assessment of diameter and pressure waveforms in the carotid artery. *Ultrasound Med. Biol.* 30(2):147–154, 2004. 14998666 10.1016/j.ultrasmedbio.2003.10.014

[34] Nabeel, P., V. R. Kiran, J. Joseph, V. Abhidev, and M. Sivaprakasam. Local pulse wave velocity: theory, methods, advancements, and clinical applications. *IEEE Rev. Biomed. Eng.* 13:74–112, 2019. 31369386 10.1109/RBME.2019.2931587

[35] Parker, K. H. Arterial tube laws and wave speeds, ArXiv Prepr. ArXiv210610061, 2021.

[36] Gasser, T. C., R. W. Ogden, and G. A. Holzapfel. Hyperelastic modelling of arterial layers with distributed collagen fibre orientations. *J. R. Soc. Interface*. 3(6):15–35, 2006. 16849214 10.1098/rsif.2005.0073 PMC1618483

[37] Xiao, N., J. Alastruey, and C. Alberto Figueroa. A systematic comparison between 1-D and 3-D hemodynamics in compliant arterial models. *Int. J. Numer. Methods Biomed. Eng.* 30(2):204–231, 2014. 10.1002/cnm.2598 PMC4337249 24115509

[38] Zhu, C., V. Vedula, D. Parker, N. Wilson, S. Shadden, and A. Marsden. svFSI: a multiphysics package for integrated cardiac modeling. *J. Open Source Softw.* 7(78):4118, 2022.

[39] Updegrove, A., N. M. Wilson, J. Merkow, H. Lan, A. L. Marsden, and S. C. Shadden. SimVascular: an open source pipeline for cardiovascular simulation. *Ann. Biomed. Eng.* 45:525–541, 2017. 27933407 10.1007/s10439-016-1762-8 PMC6546171

[40] Laurent, S., et al. Elastic modulus of the radial artery wall material is not increased in patients with essential hypertension. *Arterioscler. Thromb. J. Vasc. Biol.* 14(7):1223–1231, 1994. 10.1161/01.atv.14.7.1223 8018679

[41] Bussy, C., P. Boutouyrie, P. Lacolley, P. Challande, and S. Laurent. Intrinsic stiffness of the carotid arterial wall material in essential hypertensives. *Hypertension*. 35(5):1049–1054, 2000. 10818063 10.1161/01.hyp.35.5.1049

[42] Bantwal, A., A. Singh, A. R. Menon, and N. Kumar. Pathogenesis of atherosclerosis and its influence on local hemodynamics: a comparative FSI study in healthy and mildly stenosed carotid arteries. *Int. J. Eng. Sci.* 167:103525, 2021.

[43] Getachew, D., A. Astatkie, and K. Lemma. Diameter, vessel thickness and angle of bifurcation of the radial artery in ethiopian cadavers. *J. Morphol. Sci.* 35(02):129–135, 2018.

[44] svfsi structural documentation. https://simvascular.github.io/documentation/simcardio.html#mechanics-theory. Accessed 12 Dec 2024.

[45] Lindström, S. B., G. Satha, and A. Klarbring. Extension of Murray’s law including nonlinear mechanics of a composite artery wall. *Biomech. Model. Mechanobiol.* 14:83–91, 2015. 24817182 10.1007/s10237-014-0590-8 PMC4282710

[46] Krejza, J., et al. Carotid artery diameter in men and women and the relation to body and neck size. *Stroke*. 37(4):1103–1105, 2006. 16497983 10.1161/01.STR.0000206440.48756.f7

[47] Charlton, P. H., J. Mariscal Harana, S. Vennin, Y. Li, P. Chowienczyk, and J. Alastruey. Modeling arterial pulse waves in healthy aging: a database for in silico evaluation of hemodynamics and pulse wave indexes. *Am. J. Physiol.-Heart Circ. Physiol.* 317(5):H1062–H1085, 2019. 31442381 10.1152/ajpheart.00218.2019 PMC6879924

[48] Figueroa, C. A., I. E. Vignon-Clementel, K. E. Jansen, T. J. Hughes, and C. A. Taylor. A coupled momentum method for modeling blood flow in three-dimensional deformable arteries. *Comput. Methods Appl. Mech. Eng.* 195(41–43):5685–5706, 2006.

[49] Westerhof, N., J.-W. Lankhaar, and B. E. Westerhof. The arterial windkessel. *Med. Biol. Eng. Comput.* 47(2):131–141, 2009. 18543011 10.1007/s11517-008-0359-2

[50] Bäumler, K., et al. Fluid–structure interaction simulations of patient-specific aortic dissection. *Biomech. Model. Mechanobiol.* 19(5):1607–1628, 2020. 10.1007/s10237-020-01294-8. 31993829 10.1007/s10237-020-01294-8

[51] Moireau, P., et al. External tissue support and fluid–structure simulation in blood flows. *Biomech. Model. Mechanobiol.* 11:1–18, 2012. 21308393 10.1007/s10237-011-0289-z

[52] Geuzaine, C., and J.-F. Remacle. Gmsh: a 3-D finite element mesh generator with built-in pre-and post-processing facilities. *Int. J. Numer. Methods Eng.* 79(11):1309–1331, 2009.

[53] Gaddum, N., J. Alastruey, P. Beerbaum, P. Chowienczyk, and T. Schaeffter. A technical assessment of pulse wave velocity algorithms applied to non-invasive arterial waveforms. *Ann. Biomed. Eng.* 41:2617–2629, 2013. 23817766 10.1007/s10439-013-0854-y

[54] Chiu, Y. C., P. W. Arand, S. G. Shroff, T. Feldman, and J. D. Carroll. Determination of pulse wave velocities with computerized algorithms. *Am. Heart J.* 121(5):1460–1470, 1991. 2017978 10.1016/0002-8703(91)90153-9

[55] Bramwell, J. C., and A. V. Hill. The velocity of pulse wave in man. *Proc. R. Soc. Lond. Ser. B Contain. Pap. Biol. Character*. 93(652):298–306, 1922.

[56] MATLAB Version: 24.1.0.2537033 (R2024a). The MathWorks Inc., Natick, Massachusetts, United States, 2024. https://www.mathworks.com.

[57] Huynh, T. H., R. Jafari, and W.-Y. Chung. Noninvasive cuffless blood pressure estimation using pulse transit time and impedance plethysmography. *IEEE Trans. Biomed. Eng.* 66(4):967–976, 2018. 30130167 10.1109/TBME.2018.2865751

[58] Kjeldsen, S. E. Hypertension and cardiovascular risk: general aspects. *Pharmacol. Res.* 129:95–99, 2018. 29127059 10.1016/j.phrs.2017.11.003

[59] Verdecchia, P., et al. Sudden cardiac death in hypertensive patients. *Hypertension*. 73(5):1071–1078, 2019. 30827144 10.1161/HYPERTENSIONAHA.119.12684

[60] Xu, L., et al. Evaluation of carotid artery blood pressure waveform using a wearable ultrasound patch. In: 2023 IEEE 19th International Conference on Body Sensor Networks (BSN). IEEE, 2023, pp. 1–4.

[61] Benthin, M., P. Dahl, R. Ruzicka, and K. Lindström. Calculation of pulse-wave velocity using cross correlation—effects of reflexes in the arterial tree. *Ultrasound Med. Biol.* 17(5):461–469, 1991. 1962347 10.1016/0301-5629(91)90182-v

[62] Mynard, J. P., and A. Kondiboyina. Wave reflection in the arterial tree. In: Textbook of Arterial Stiffness and Pulsatile Hemodynamics in Health and Disease, edited by J. A. Chirinos. Academic Press, 2022, pp. 169–194. 10.1016/B978-0-323-91391-1.00011-X.

[63] Lillie, J. S., A. S. Liberson, and D. A. Borkholder. Improved blood pressure prediction using systolic flow correction of pulse wave velocity. *Cardiovasc. Eng. Technol.* 7:439–447, 2016. 27730533 10.1007/s13239-016-0281-y

[64] Manoj, R., S. Ponkalaivani, P. Nabeel, J. Joseph, et al. Effect of fiduciary point choice on pulse wave velocity-based cuffless pulse pressure estimation: ex-vivo study. In: 2023 IEEE International Symposium on Medical Measurements and Applications (MeMeA). IEEE, 2023, pp. 1–6.

[65] Jimenez, R., et al. Resonance sonomanometry for noninvasive, continuous monitoring of blood pressure. *PNAS Nexus*. 3(7):pgae252, 2024. 39081785 10.1093/pnasnexus/pgae252 PMC11287871

[66] Zhou, S., et al. Clinical validation of a wearable ultrasound sensor of blood pressure. *Nat. Biomed. Eng.* 2024. 10.1038/s41551-024-01279-3. 39567702 10.1038/s41551-024-01279-3

